# Adults following open esophageal atresia repair: evaluating long-term musculoskeletal and pulmonary outcomes using insights from real-time MRI

**DOI:** 10.1038/s41390-025-04568-y

**Published:** 2025-12-03

**Authors:** Ophelia Aubert, Martin Lacher, Jens Frahm, Dirk Voit, Anke Widenmann, Franz Wolfgang Hirsch, Maciej Rosolowski, Daniel Gräfe

**Affiliations:** 1 Department of Pediatric Surgery, University Hospital Leipzig, Leipzig, Germany; 2 Department of Pediatric Surgery, University Medical Center Mannheim, University of Heidelberg, Mannheim, Germany; 3 Biomedical NMR, Max Planck Institute for Multidisciplinary Sciences, Göttingen, Germany; 4 Patient Organization for Esophageal Diseases KEKS e.V., Stuttgart, Germany; 5 Department of Pediatric Radiology, University Hospital Leipzig, Leipzig, Germany; 6 Institute for Medical Informatics, Statistics and Epidemiology, Leipzig University, Leipzig, Germany

## Abstract

**Background:**

EA survivors face long-term morbidity after thoracotomy with adult outcomes largely uninvestigated. This study assessed musculoskeletal and pulmonary outcomes in adults who underwent neonatal thoracotomy for EA using real-time magnetic resonance imaging (rt-MRI).

**Methods:**

Between 09/2022 and 02/2023, 30 adult EA patients and 22 healthy controls underwent rt-MRI to evaluate chest wall motion, lung volumes, and anatomical deformities. Thoracic cross-sectional area (CSA), lung volumes, and rib/spinal abnormalities were analyzed using a 3 Tesla MRI system.

**Results:**

Rib adhesions and fusions were significantly more frequent in EA patients (90%) than in controls (*p* < 0.01). Scoliosis was present in 43% of patients, with many cases moderate to severe (*p* < 0.05). EA patients showed reduced thoracic CSA and lung volumes on the operated side (*p* < 0.01) and significantly impaired chest wall motility (*p* < 0.05). Scoliosis prevalence was higher in adults (43%) than in children (14%) (*p* < 0.05).

**Conclusion:**

This study demonstrates the significant long-term musculoskeletal and pulmonary sequelae in adults following open EA repair, with the pioneering application of rt-MRI. These findings emphasize the risks associated with open surgery for EA, underscoring the need for long-term, multidisciplinary follow-up and preventive strategies.

**Impact:**

- This is the first study to use real-time MRI to assess dynamic thoracic function in adults after open repair of esophageal atresia (EA), revealing significant long-term musculoskeletal and pulmonary impairments.
- Findings include high rates of scoliosis, rib anomalies, and reduced chest wall motility and lung volume on the operated side.
- Results underscore the need for long-term, multidisciplinary follow-up and inform future strategies to reduce morbidity after neonatal thoracotomy.

## Introduction

The survival rate of children with esophageal atresia (EA) has significantly improved over the past decades, resulting in a growing population of adults requiring long-term follow-up care.^1^ However, structured transition programs and systematic long-term care protocols for these patients are often lacking, leading to undiagnosed morbidity. The vast majority of adult patients were treated with an open surgical approach, as thoracoscopic repair of EA was only introduced approximately 25 years ago.^2^ Even today, only a minority of children undergo surgery via thoracoscopy.^3^ Thoracotomy performed during the neonatal period is associated with musculoskeletal complications and restrictive ventilatory defects, which contribute to long-term morbidity.^4–6^ Nevertheless, long-term studies evaluating musculoskeletal deformities and pulmonary function and volumes in adults with EA remain limited.^3^

Using real-time magnetic resonance imaging (rt-MRI), we have recently demonstrated that pediatric EA patients exhibit significantly smaller lung and thoracic volumes on the operated side, along with a higher incidence of anatomical deformities.^6^ Rt-MRI is an advanced imaging technique that provides dynamic, real-time visualization of anatomical structures in motion, allowing for a detailed assessment of both static and functional abnormalities. It offers several advantages, including the absence of ionizing radiation, the need for contrast medium, or reliance on patient compliance, while providing superior functional evaluation compared to conventional diagnostic methods.^4,7–9^

In this study, we prospectively evaluated musculoskeletal and pulmonary outcomes in a cohort of adult EA patients using the novel rt-MRI technique.^6,9^

## Methods

### Patient cohort

This prospective study was conducted between September 2022 and February 2023. IRB approval (0341/21-ek) and informed consent from all study participants were obtained. Recruitment was based on a query of the hospital’s internal patient database and advertisements through a nationwide German patient organization for esophageal diseases (KEKS e.V., Stuttgart, Germany).

Inclusion criteria were an age of over 18 years and a history of EA repair in the neonatal period with right-sided surgical access. Exclusion criteria were contraindications to MRI (e.g., incompatible metal implants, pacemakers, or claustrophobia), excessive movement during MRI, and situs abnormalities of the chest. Additionally, healthy participants with a similar age and sex distribution were recruited as controls. No sex differences were expected.

### Real-time MRI technique

All examinations were performed using a 3 Tesla MRI system (Prisma fit, Siemens Healthcare, Erlangen, Germany) equipped with an 18-channel body receive coil and spine receive coil. The rt-MRI techniques applied in this study have been previously described, and our group has extensive experience with this method, which enables simultaneous morphological and functional evaluation of the thorax during free breathing.^6,9–14^

Briefly, the technique is based on a highly undersampled radial gradient echo sequence, followed by joint nonlinear inverse reconstruction of serial images and their coil sensitivity maps. This approach allows for a true acquisition time of up to 20 ms per slice. Ultra-fast image acquisition can be used for either high temporal dynamics on a single cross-section or rapid acquisition of multiple partially overlapping slices by introducing a small slice shift with each image.^9,11^ As a result, an entire volume can be scanned within a few seconds (volume coverage technique), producing high-speed images free from motion artifacts.^12,13^

The rt-MRI protocol consisted of three components:

1. Chest wall motion assessment during breathing: Three parallel, synchronously acquired axial slices with high temporal dynamics (proton density-weighted (PDw), temporal resolution of 34 ms per frame, slice thickness 6 mm, in-plane resolution 1.4 × 1.4 mm).

2. Volume coverage (VC) sequences for lung volume determination: Short breath-hold maneuvers in maximal inspiration and expiration (PDw: temporal resolution of 57 ms per image, slice increment of 2.5 mm per image, corresponding to approximately 8 s measuring time for a 35 cm volume; slice thickness 5 mm, in-plane resolution 1.0 × 1.0 mm).

3. Anatomical assessment: Coronal and paracoronal VC sequences for the evaluation of thoracic wall structure, vertebral malformations, and rib pathologies. A rib adhesion was defined as an approximation of two adjacent ribs compared to the contralateral side, as well as to neighboring ribs. A rib fusion was defined as a partial osseous merging of two or more adjacent ribs. (PDw as above; T2/T1-weighted: temporal resolution of 99 ms per image, slice increment 0.88 mm per image, corresponding to 8.7 mm volume coverage; slice thickness 3.5 mm, in-plane resolution 1.0 × 1.0 mm) (Video 2).

### MRI analysis

Thoracic cross-sectional area (CSA) measurements were performed using a standard PACS system (IDS7, Sectra AB, Linköping, Sweden). The inner border of the thoracic wall was outlined, and the right and left thoracic CSAs were measured, separated by a line drawn between the midpoints of the sternum and the vertebral body.^6^ Lung volume measurements were conducted in Intellispace Portal v12 (Philips, Best, The Netherlands). Using the intensity threshold tool, signal-free lung portions were measured separately for the right and left sides during maximal inspiration and expiration. Larger vessels, particularly near the hilum, were excluded from the measurements. The accuracy of this method has been previously validated using phantom models.^6^ Anatomical assessments of the thoracic wall, ribs, and spine were performed qualitatively using the IDS7 PACS system.

### Statistical analysis

The association between CSA and VC measurements and different groups (patients vs. controls, adult group vs. pediatric group) or the number of surgeries was examined using linear regression. The outcome variable was the measurement on the right side, specifically, csa_right_insp_, csa_right_exsp,_ (csa_right_insp_ - csa_right_exsp_), vc_right_insp_, vc_right_exsp,_ or (vc_right_insp_ - vc_right_exsp_). Covariates included the corresponding measurement on the left side (to enable comparison with the right side and serve as a self-control for person-specific factors), the variable of interest (group or the number of surgeries), and their interaction. Two-degree-of-freedom tests were conducted to assess the overall effect of the variable of interest, with Holm-Bonferroni correction applied to control the familywise error rate. The interaction was evaluated only if the overall effect was significant. Age was compared between patients and controls using Mann–Whitney U test. Associations between dichotomous variables were evaluated with Chi-squared tests. *P* values < 0.05 were considered statistically significant. All statistical analyses were performed using R Studio v2023.09.1 + 494 (Posit Software, Boston, MA).

## Results

### Demographic data

We recruited 31 patients and 22 healthy controls. One patient, who underwent a two-stage EA repair involving a combined laparotomy and cervical approach, was excluded from the study. Consequently, a total of 30 patients (median age 28.7 years, IQR 23.0–40.0 years; 17 female) and 22 healthy volunteers (median age 31.3 years, IQR 25.6–36.9 years; 15 female) were included in this prospective study (Table 1). All patients had undergone right-sided thoracotomy.

**Table 1:** Demographic dataand anatomical abnormalities of EA patients and controls.

	EA Patients (*n* = 30)	Controls (*n* = 22)	*p* value
Age (y)	28.7 [23.0–40.0]	31.3 [25.6–36.9]	ns
Gender (m: w)	13 : 17	7 : 15	ns
EA-type (%)			
A	5 (17%)	NA	
B	2 (7%)		
C	21 (70%)		
D	1 (3%)		
Unknown	1 (3%)		
Patients ≥ 2 thoracotomies, *n* (%)	7 (24%)	NA	
Number of thoracotomies (*n*)	1.3 ± 0.8	NA	
Rib fusions	15 (50%)	0 (0%)	****
Rib adhesions	12 (40%)	0 (0%)	***
Scoliosis	13 (43%)	0 (0%)	***

Median and interquartile range is given, ***p < 0.001, ****p ≤ 0.0001.

The majority of patients (21/30, 70%) had EA type C, while 5/30 (17%) had EA type A, 2/30 (7%) had EA type B, and 1/30 (3%) had EA type D. The type of EA was unknown for one patient. The mean birth weight was 2635 ± 494 g, and the mean length at birth was 48.2 ± 3.0 cm. No significant differences in age (*p* = 0.47) or sex (*p* = 0.58) were observed between patients and the control group.

### Anatomical abnormalities

**Fig. 1:**
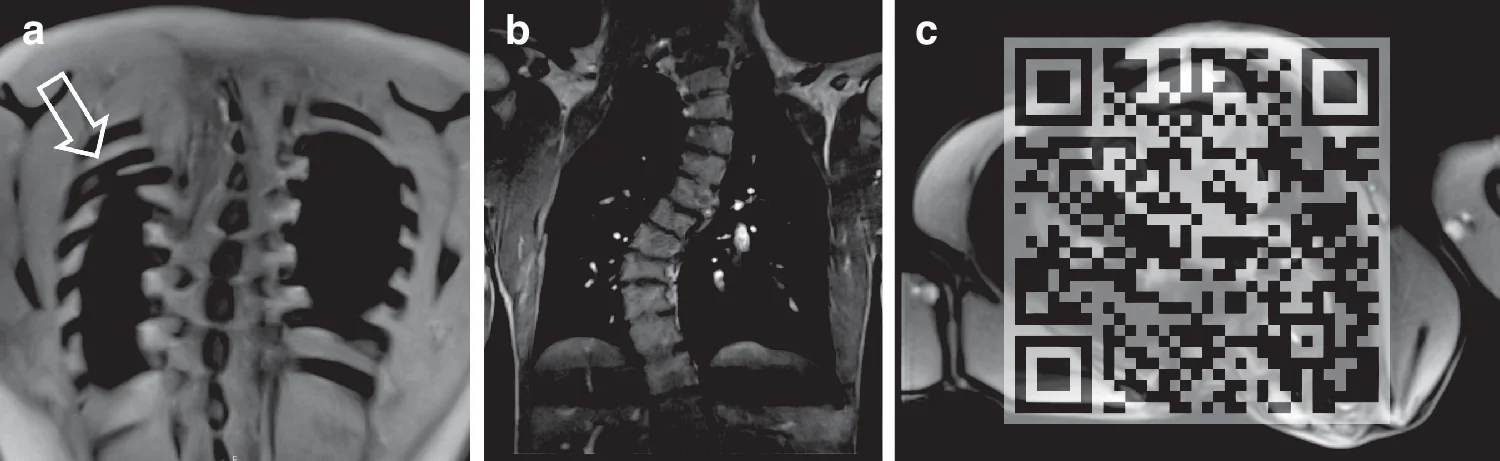
Anatomical deformities after open EA repair. **a** Fusion of Rib 4 and 5 (arrow) and **b** scoliosis in a 29- and 27-year-old patient, respectively, following open EA repair. **c** Real-Time MRI of a patient with severe vertebral and chest wall anomaly in 20-year-old EA patient. (https://pedz.de/supplementals/ea-adult/video1.mp4).

Adult EA patients exhibited significantly more rib adhesions (12/30, 40%) and rib fusions (15/30, 50%) compared to healthy controls (*p* < 0.01 and *p* < 0.001, respectively; Table 1 and Fig. 1a). All abnormalities were located on the right side, predominantly involving ribs 3, 4, or 5.

Scoliosis was observed in 13/30 (43.3%) EA patients (*p* < 0.01; Table 1 and Fig. 1b). Among these, 6/13 (46%) had grade I scoliosis (<25°), while 4/13 (31%) had moderate grade II scoliosis (25°–40°), and 3/13 (23%) had severe grade III scoliosis (>40°). One patient presented with a butterfly-shaped vertebral body in the mid-thoracic spine, along with severe left hemithorax deformity characterized by a 180° rib rotation and severe scoliosis, despite multiple spine distractions and implantation of a vertical expandable prosthetic titanium rib device (Fig. 1c). No other patient underwent surgical intervention for scoliosis of chest wall deformity.

### Thoracic dimensions

**Fig. 2:**
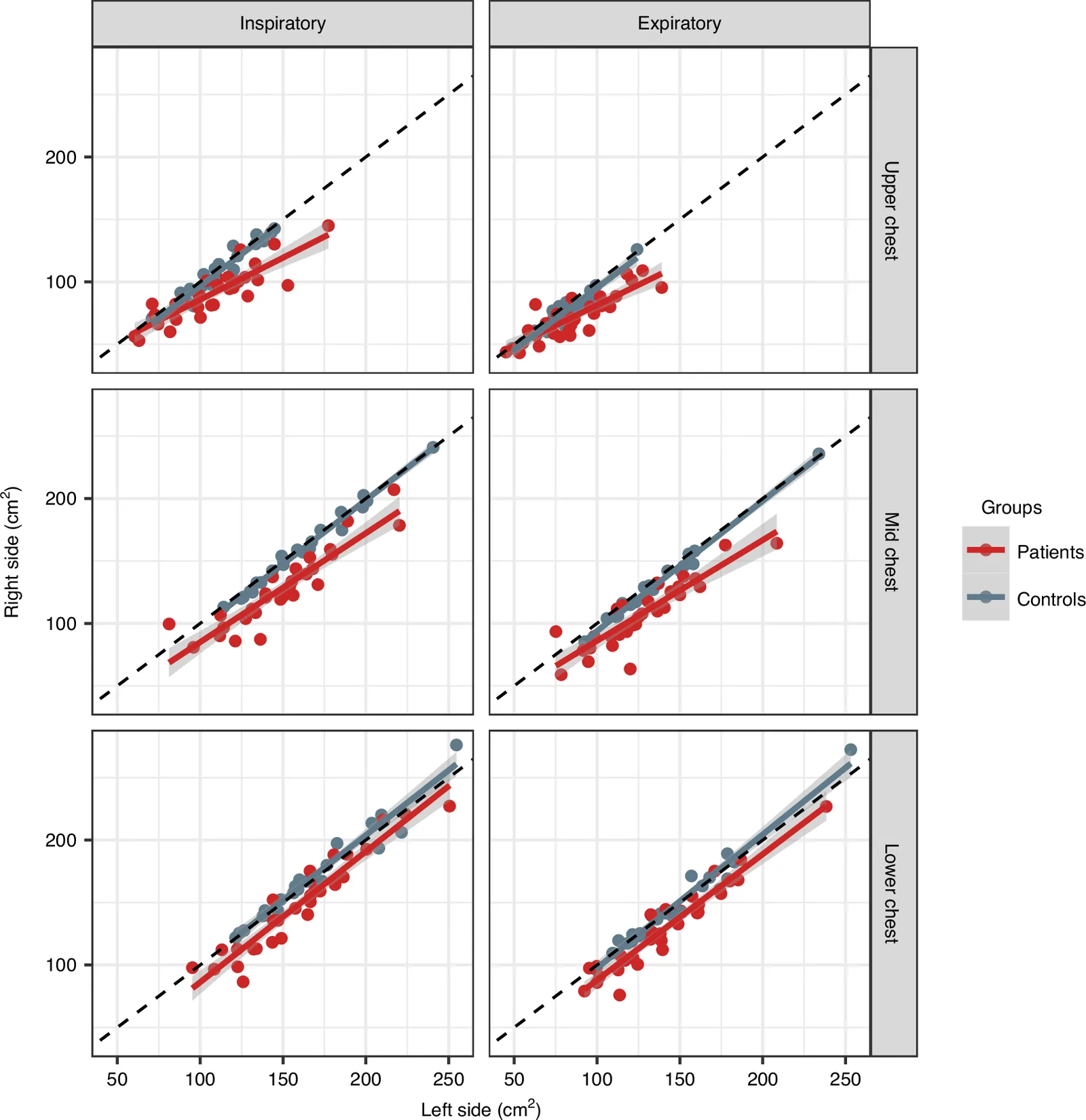
Cross-sectional areas of the right hemithorax relative to the left in patients with esophageal atresia compared to healthy controls. Measurements were performed at three different thoracic levels during both inspiration and expiration (dotted line: bisector).

**Fig. 3:**
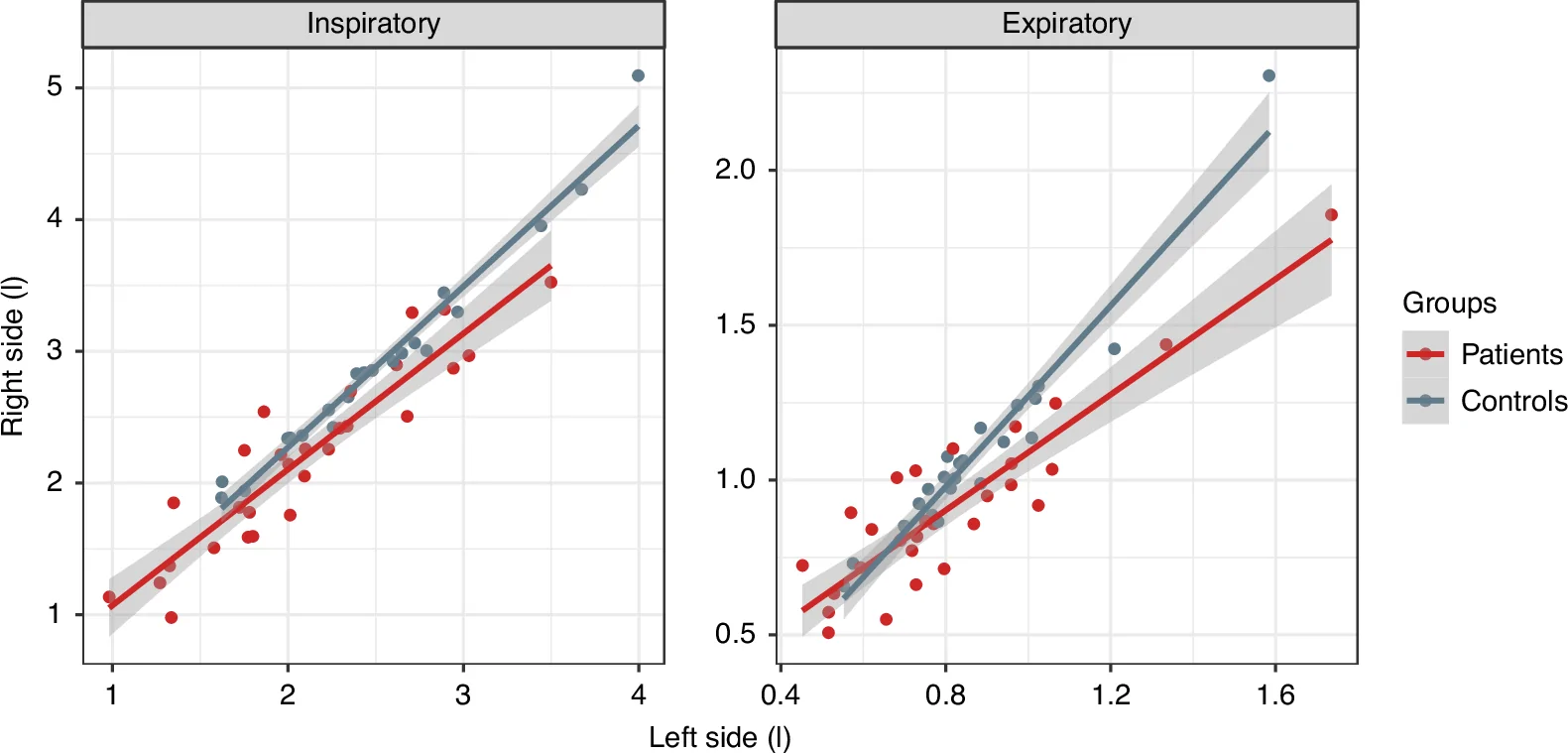
Lung volume of the right hemithorax relative to the left in patients after esophageal atresia repair compared to healthy controls. Measurements were performed during both maximal inspiration and expiration.

The CSA of the right hemithorax relative to the left was significantly smaller at all three investigated levels compared to healthy controls (*p* < 0.001 for each level; Fig. 2). The lung volume of the right hemithorax, relative to the left lung, was significantly smaller in patients with esophageal atresia (*p* < 0.01 for each comparison; Fig. 3). No significant correlation was found between lung volumes and the number of thoracotomies (*p* = 0.94).

### Chest wall motility

**Fig. 4:**
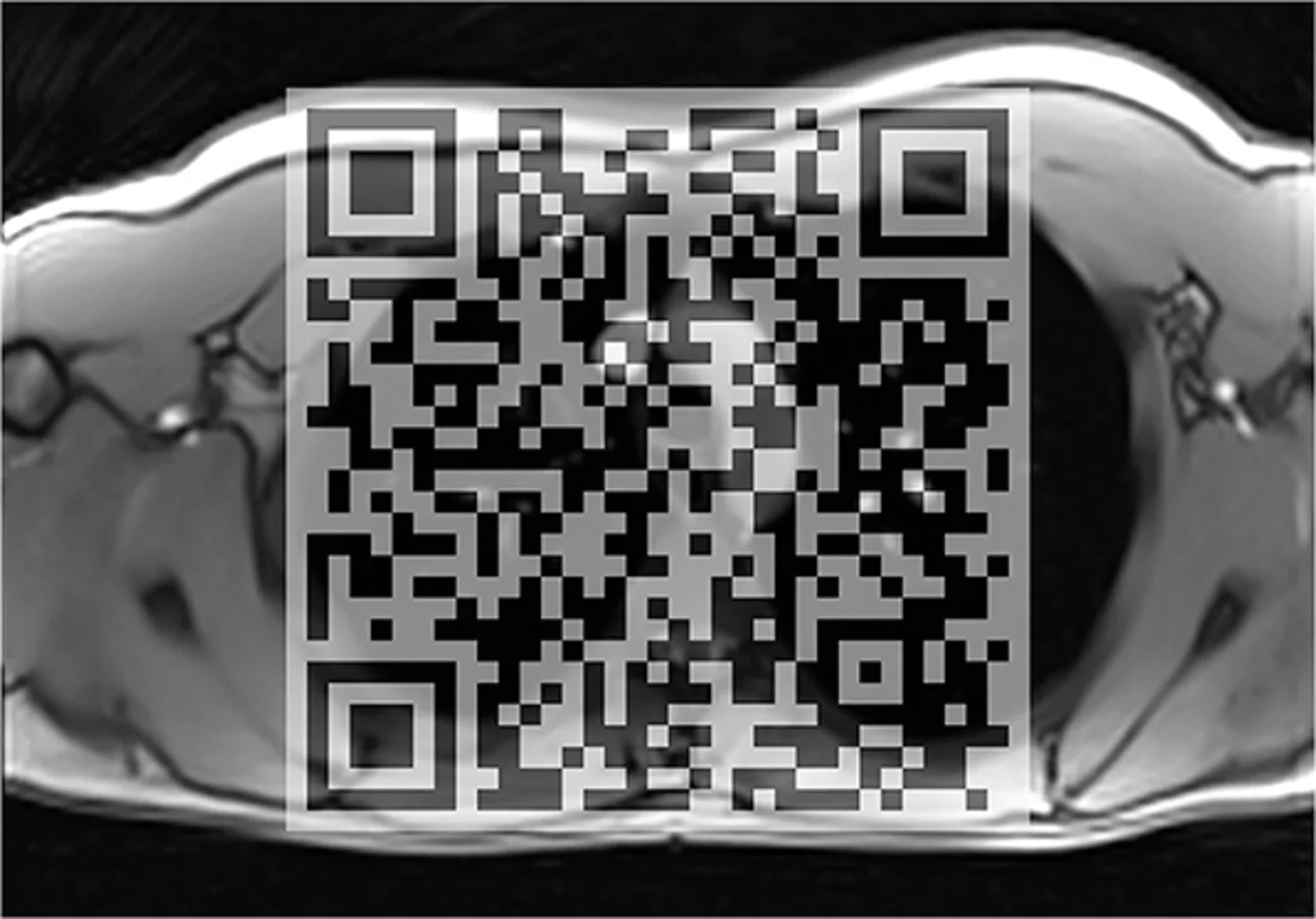
Real-time MRI of a 24-year-old patient following neonatal repair of esophageal atresia. In the axial plane, reduced motion of the right hemithorax at the level of a rib fusion (Ribs 4 and 5) is clearly visible. (https://pedz.de/supplementals/ea-adult/video1.mp4).

**Fig. 5:**
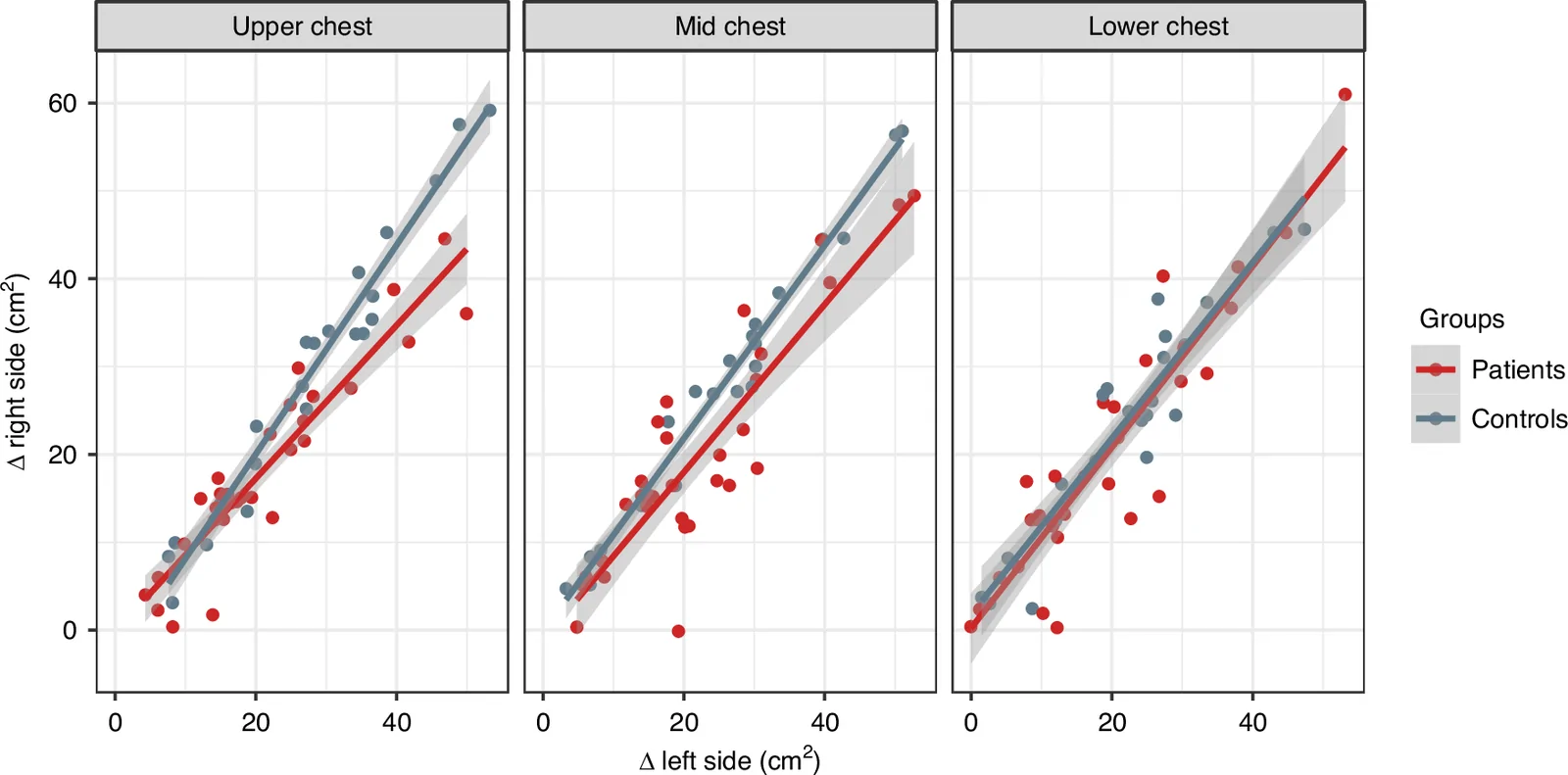
Dynamic changes in cross-sectional areas during forced breathing as a measure of chest wall motility in patients after esophageal atresia repair compared to healthy controls. The relative area of the right hemithorax to the left hemithorax was measured at three distinct thoracic levels.

**Fig. 6:**
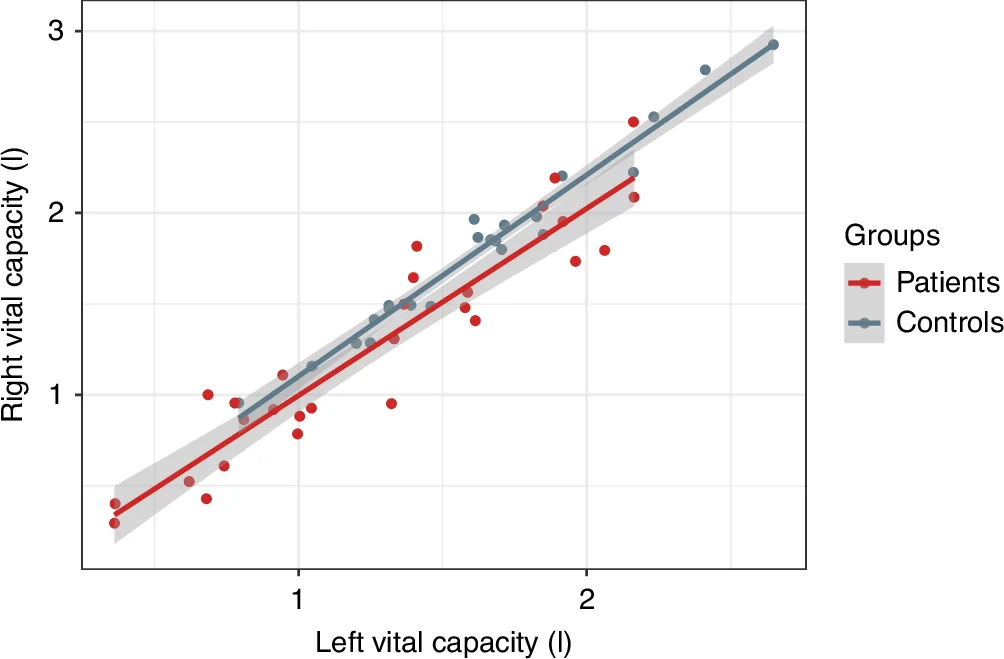
Changes in lung volume during forced breathing in patients after esophageal atresia repair compared to healthy controls. The relative volume change of the right lung compared to the left lung is presented.

Qualitative assessment of the rt-MRI videos showed impaired right-sided chest wall motility during forced breathing in EA patients (Fig. 4). Changes in thoracic CSA and lung volume during respiration were measured to assess chest wall motility and respiratory reserve. Motility of the right hemithorax in EA patients was reduced compared to the left side at the upper and middle level of the chest (*p* < 0.05), but not at the lower thoracic level (*p* = 0.79) (Fig. 5). This resulted in a lower lung vital capacity of the right side, compared to the left (*p* < 0.01) in comparison to healthy controls (Fig. 6). Motility of the right hemithorax was significantly correlated to the number of thoracotomies at the middle and lower chest level (*p* < 0.05 and *p* < 0.01 respectively.

### Adults vs. children

Results from adult EA patients were compared to a previously studied pediatric cohort⁶. The right-to-left thorax volume in adult patients showed no significant difference compared to pediatric patients (*p* = 0.46 for inspiration and *p* = 0.19 for expiration). The prevalence of rib fusions and adhesions was also similar between adults and children who underwent open surgery (rib fusions + adhesions: adults 90% vs. children 79%, *p* = 0.34). However, scoliosis was significantly more common in adults than in children (43% vs. 14%, *p* < 0.05), suggesting that the severity of scoliosis may progress over time.

## Discussion

In this prospective study, we demonstrated the long-term sequelae of open EA repair in an adult patient cohort using functional high-resolution rt-MRI. Importantly, the investigation did not require radiation, sedation, or contrast medium, making it a safe and effective method for long-term assessment.

Structured transition-of-care programs for adults with congenital malformations are often lacking, despite the persistence or worsening of issues originating in childhood.^15^ While gastrointestinal problems such as gastroesophageal reflux disease or swallowing difficulties frequently dominate doctor-patient interactions, long-term musculoskeletal morbidity should not be overlooked. A recent systematic review of long-term outcomes and transitional care in adolescents and adults, encompassing 16 studies with 830 patients, reported musculoskeletal outcomes for only 74 patients.^16^ Of these, a third exhibited scoliosis, and only 1% had rib fusions. In our study, scoliosis was observed in almost half of the patients, with many cases classified as moderate or severe - findings consistent with previous research in EA patients.^4,17,18^ Furthermore, rib fusions or adhesions, known risk factors for scoliosis, were present in 90% of our cohort, significantly higher than what has been reported in the aforementioned systematic review or studies using conventional X-ray imaging.^16,19^ This again confirms that rt-MRI is more sensitive in detecting postoperative rib abnormalities. Additionally, the incidence of scoliosis after open EA repair was significantly higher in our adult cohort compared to our previous study in the pediatric population, indicating that musculoskeletal deformities develop or worsen over time.^6^ These findings highlight the substantial trauma associated with neonatal thoracotomy and the progressive deterioration of outcomes decades after the initial surgery. They also underscore the inadequacies in musculoskeletal follow-up and suggest that the true prevalence of anatomical deformities may be underestimated when patients are not systematically assessed.

To evaluate thoracic development, we measured the CSA at three distinct levels (upper, mid, and lower chest) and adjusted the right thoracic CSA for its value on the left using regression. Adult EA patients who underwent open repair exhibited significantly reduced right CSA adjusted for the left at all levels compared to controls. These findings suggest compromised thoracic wall development on the operated side, with the greatest impact localized to the incision site. Furthermore, the differences were statistically significant during both inspiration and expiration, indicating that the thoracic abnormalities affect both static (expiration) and functional (inspiration) states.

Reduced right-sided thoracic CSA was associated with decreased lung volumes in adult EA patients. It is well established that EA patients experience significant respiratory morbidity, including frequent early-life respiratory infections and persistent or emerging conditions such as bronchiectasis in adulthood.^15,20^ We assessed lung volumes in a similar way as the CSA, i.e., by adjusting the right lung volume for the left using regression, providing the first report of side-specific lung volumes in an adult EA patient cohort. Adults post-EA repair demonstrated significantly smaller right lung volume adjusted for the left compared to controls. We did not find the difference in thoracic and lung volumes observed during both expiration and inspiration to be more pronounced in adults than in children, suggesting that thoracic and lung underdevelopment on the operated side remains stable over time. Nevertheless, this may contribute to the persistence of respiratory morbidity and be related to the high incidence of rib fusions, which are significant risk factors for both scoliosis and restrictive ventilatory defects.^19^

In contrast to our findings in the pediatric population, the number of thoracotomies was not found to be a significant risk factor for reduced right-sided thoracic CSA and lung volume. This may be related to a smaller sample size in this study or a lower number of included patients with multiple thoracotomies (adults 24% vs. children 43%). However, our data suggest an association between the number of surgeries and altered chest wall motility, with differences in left- and right-sided movement between patients with single and multiple thoracotomies. Furthermore, qualitative analysis of rt-MRI videos often indicated reduced right-sided movement in many patients. Our quantitative analysis confirmed these observations, showing that chest wall motility in EA patients was reduced on the right side at the upper and middle thoracic levels compared to the left. This was accompanied by a lower right-sided lung vital capacity, suggesting functional respiratory impairments. Taken together, these findings suggest that localized restrictions in thoracic expansion contribute to altered respiratory mechanics in adults who underwent neonatal open EA repair and correlate with the number of thoracotomies.

Our prospective study provided an unprecedented level of detail about the anatomy and function of the chest decades after open EA repair, leveraging advanced rt-MRI technology. To our knowledge, this is the first study to use this technique in an adult EA cohort. Our findings reveal that nearly all adult patients exhibit musculoskeletal abnormalities, with half presenting scoliosis and a quarter experiencing moderate to severe scoliosis - rates significantly higher than in our previous pediatric cohort.^6^ We acknowledge the limitations of our study, particularly the small patient cohort and possible participation bias. Patients with ongoing morbidity may have been more inclined to participate, and the results observed here could be worse than those seen in the average adult EA patient. Additionally, we were only able to access operative records for approximately half of patients, and available records oftentimes did not outline the specific surgical approach. Thus, we were unable to examine known confounders such as muscle-sparing approaches, size and site of incision, or whether extra-pleural access was achieved, factors known to influence musculoskeletal deformities.^21,22^ However, controlling for these confounders may uncover significant differences in long-term morbidity between approaches, suggesting that the impact of thoracotomy is less severe than generally assumed. Nevertheless, these results underscore the necessity of comprehensive, lifelong follow-up programs for EA patients, involving a multidisciplinary team including pediatric surgeons, pulmonologists, orthopedic surgeons, and physiotherapists.^15^ Furthermore, they emphasize the need to identify and implement preventive strategies, such as targeted physiotherapy or sports programs, to minimize the risk of scoliosis and muscular deformities in the long term, thereby improving quality of life for EA patients.

Despite being available for 25 years with well-documented benefits, thoracoscopic repair remains underutilized, with only a minority of patients undergoing this minimally invasive procedure.^3^ Our findings highlight the urgent need to reassess current patient care practices, particularly for those undergoing open repair, and to explore the potential advantages of centralizing care for these rare conditions. Further studies are needed to examine the impact of surgical caseloads on outcomes, providing insights to enhance patient management strategies.

## Conclusion

This study highlights significant long-term musculoskeletal and pulmonary complications in adults following open esophageal atresia repair, including rib fusions, scoliosis, reduced right-sided thoracic and lung development, as well as impaired right-sided chest wall motility and lung vital capacity. The findings underscore the need for multidisciplinary follow-up care, preventive strategies like physiotherapy, and the advantages of minimally invasive thoracoscopic repair. Centralized care and further research are essential to improve outcomes for adult EA survivors.

## Data Availability

Data will be made available by the corresponding author upon reasonable request.
